# Abscopal Effect of Radiotherapy Enhanced with Immune Checkpoint Inhibitors of Triple Negative Breast Cancer in 4T1 Mammary Carcinoma Model

**DOI:** 10.3390/ijms221910476

**Published:** 2021-09-28

**Authors:** Haa-Na Song, Hana Jin, Jung-Hoon Kim, In-Bong Ha, Ki-Mun Kang, Hoon-Sik Choi, Ho-Jin Jeong, Min-Young Kim, Hye-Jung Kim, Bae-Kwon Jeong

**Affiliations:** 1 Division of Hemato-Oncology, Department of Internal Medicine, Gyeongsang National University of Medicine and Gyeongsang National University Hospital, Jinju 52727, Korea; songhaana@gnu.ac.kr (H.-N.S.); willgraham@naver.com (J.-H.K.); 2 Institute of Health Science, Gyeongsang National University, Jinju 52727, Korea; hanajin.kr@daum.net (H.-N.J.); nicehib@gnuh.co.kr (I.-B.H.); jsk92@gnu.ac.kr (K.-M.K.); radignuh@gnuh.co.kr (H.-S.C.); jeong3023@gnu.ac.kr (H.-J.J.); hyejungkim@gnu.ac.kr (H.-J.K.); 3 Biomedical Research Institute, Gyeongsang National University Hospital, Jinju 52727, Korea; 4 Department of Pharmacology, School of Medicine, Gyeongsang National University, Jinju 52727, Korea; 5 Department of Radiation Oncology, Gyeongsang National University of Medicine and Gyeongsang National University Hospital, Jinju 52727, Korea; 6 Department of Radiation Oncology, Gyeongsang National University Changwon Hospital, Gyeongsang National University College of Medicine, Changwon 51472, Korea; 7 Division of Endocrinology, Department of Internal Medicine, Gyeongsang National University of Medicine and Gyeongsang National University Hospital, Jinju 52727, Korea; minyung000@hanmail.net

**Keywords:** abscopal effect, radiotherapy, immune checkpoint inhibitor, triple-negative breast cancer

## Abstract

Local radiotherapy (RT) is important to manage metastatic triple-negative breast cancer (TNBC). Although RT primarily reduces cancer cells locally, this control can be enhanced by triggering the immune system via immunotherapy. RT and immunotherapy may lead to an improved systemic effect, known as the abscopal effect. Here, we analyzed the antitumor effect of combination therapy using RT with an anti-programmed cell death-1 (PD-1) antibody in primary tumors, using poorly immunogenic metastatic mouse mammary carcinoma 4T1 model. Mice were injected subcutaneously into both flanks with 4T1 cells, and treatment was initiated 12 days later. Mice were randomly assigned to three treatment groups: (1) control (no treatment with RT or immune checkpoint inhibitor (ICI)), (2) RT alone, and (3) RT+ICI. The same RT dose was prescribed in both RT-alone and RT+ICI groups as 10Gy/fx in two fractions and delivered to only one of the two tumor burdens injected at both sides of flanks. In the RT+ICI group, 200 µg fixed dose of PD-1 antibody was intraperitoneally administered concurrently with RT. The RT and ICI combination markedly reduced tumor cell growth not only in the irradiated site but also in non-irradiated sites, a typical characteristic of the abscopal effect. This was observed only in radiation-sensitive cancer cells. Lung metastasis development was lower in RT-irradiated groups (RT-only and RT+ICI groups) than in the non-irradiated group, regardless of the radiation sensitivity of tumor cells. However, there was no additive effect of ICI on RT to control lung metastasis, as was already known regarding the abscopal effect. The combination of local RT with anti-PD-1 blockade could be a promising treatment strategy against metastatic TNBC. Further research is required to integrate our results into a clinical setting.

## 1. Introduction

Triple-negative breast cancer (TNBC) simultaneously lacks the expression of three different receptors: the estrogen receptor, progesterone receptor, and human epidermal growth factor receptor 2 (HER2) [1]. TNBC accounts for approximately 10–15% of all breast cancers, appears to have a more aggressive course than other breast cancers, and is more likely to spread beyond the breast at diagnosis and is more likely to recur distantly after treatment [2,3,4]. Despite its poor prognosis, TNBC is heterogeneous with regard to individual patient outcomes. Several efforts have been made to improve the prognosis of TNBC, including surgery, conventional chemotherapy, and radiotherapy (RT); however, they have been less effective [5]. Recently, poly ADP-ribose polymerase (PARP) inhibitors have been shown to be a promising strategy for the treatment of TNBC, especially when associated with homologous recombination deficiency [6].

High levels of tumor-infiltrating lymphocytes, increased expression of programmed cell death-1 (PD-1), and a high tumor mutational burden suggests that immunotherapy may be a viable treatment strategy [7,8]. Pembrolizumab is a highly selective, humanized monoclonal IgG4-kappa isotype antibody against PD-1. Anti-PD-1 antibodies can reverse negative immune regulatory signaling pathways in cytotoxic T cells. Previous studies have shown that immunotherapy including pembrolizumab or atezolizumab is well tolerated in combination with chemotherapy, prolonging progression-free survival in programmed death ligand-1 (PD-L1)-positive metastatic TNBC patients [9,10]. Other clinical trials have shown that pembrolizumab monotherapy has a response rate of 4.7% in previously treated patients with TNBC regardless of PD-L1 status [11]. Despite these successes, the objective response rates of these treatment strategies remain disappointing, and many responders eventually progressed during treatment. In an effort to overcome the limitations of immunotherapy in cancer treatment, previous studies have shown that combining RT can stimulate immune responses and enhance synergistic effects [12,13,14]. RT primarily damages the DNA of cancer cells locally; it also changes the tumor microenvironment by generating local inflammatory reactions and enhancing tumor cell recognition by the host’s immune system. These local processes can be enhanced by triggering the immune system using immunotherapy [15,16]. RT-induced cancer cell damage exposes tumor-specific antigens to the immune system through a process called immunogenic cell death [17]. This process leads to improved priming and activation of cytotoxic T cells [18]. Furthermore, RT leads to the release of T-cell-attracting chemokines and the upregulation of surface receptors, which makes tumor cells more vulnerable to T-cell-mediated cell killing. Thereby, the combination of RT and immunotherapy may even lead to an improved systemic effect, also known as the “abscopal effect” (ab scopus: on a distant site), where the immune system initiates tumor regression in distant non-irradiated sites more efficiently [17,19,20].

The 4T1 tumor is mouse-derived poorly immunogenic mammary carcinoma cell lines and shares several characteristics of human breast cancer. The 4T1 mammary carcinoma can spontaneously metastasize from the primary mammary tumor to multiple distant sites, so that makes it a suitable experimental animal model for human breast cancer [21]. 

In this study, we aimed to demonstrate the abscopal effect in a preclinical model of metastatic breast cancer using 4T1 tumor and to evaluate whether combined anti-PD-1 antibody and hypofractionated RT with ablative doses could inhibit both irradiated and non-irradiated (primary and secondary, respectively) tumor growth.

## 2. Results

### 2.1. Combined RT with ICI Enhances The Abscopal Effect

**Figure 1:**
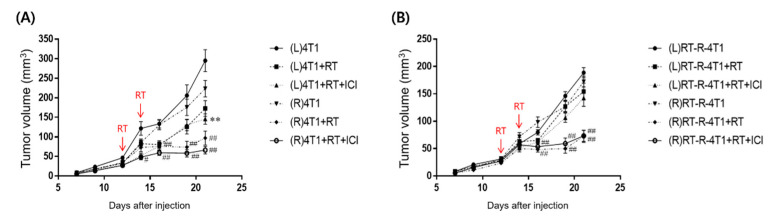
Combined PD- 1 blockade therapy and RT enhanced antitumor abscopal effects. Six-week-old female balb/c nude mice were injected with 5 × 104 of 4T1 (**A**) or RT-R-4T1 (**B**) cells into the right flank (R, primary; irradiated) and left flank (L, secondary; non-irradiated) (n = 4~6). From day 7 after inoculation, tumor volumes were measured 3 times a week until day 21. Arrows indicate the points at which RT was delivered. ** *p* < 0.01 compared to the secondary (L) tumor of non-treated 4T1 or RT-R-4T1-injected group; ^#^ *p* < 0.05, ^##^ *p* < 0.01 compared to the primary (R) tumor of non-treated 4T1 or RT-R-4T1-injected group.

The results in Figure 1A show that the combination of RT and ICI treatment resulted in a three-fold reduction (*p* < 0.01) in primary and secondary tumors. 

However, in tumors obtained using radiation-resistant cell lines, the primary tumor volume decreased, while the secondary tumor volume increased after RT and ICI treatment (Figure 1B). Therefore, clinically relevant abscopal effects were observed only in tumors obtained from radiation-sensitive cancer cell lines. 

### 2.2. Antitumor Effect in Lung Metastasis after Treatment with RT and ICI

**Figure 2:**
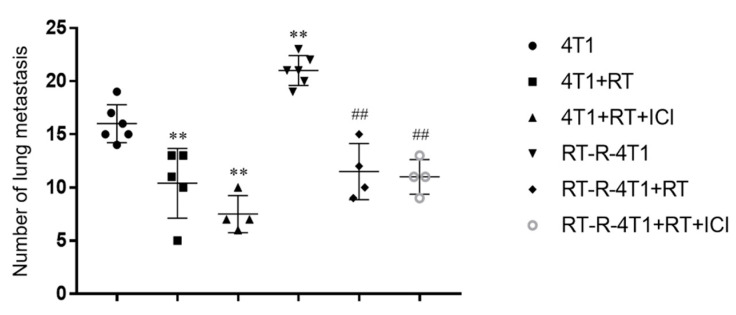
Combined PD-1 blockade therapy and RT reduced lung metastasis of breast tumor. Six-week-old female balb/c nude mice were injected as described above (n = 4–6). On day 21 after inoculation, the mice were sacrificed, and then the incidence of lung metastasis was measured. ** *p* < 0.01 compared to the 4T1-injected group; ^##^ *p* < 0.01 compared to the RT-R-4T1-injected group.

To demonstrate the effects of RT and ICI on the prevention of lung metastasis, all mice were sacrificed on day 21 post-tumor implantation, and the number of surface metastatic foci was counted under a dissecting microscope. The median number of lung metastasis was lower in all treated animals than in the controls (Figure 2). A decrease in lung metastasis was observed when RT and ICI were used in combination or RT alone. However, there was no abscopal effect of ICI on RT to control lung metastasis. 

### 2.3. Effect of RT and ICI Treatment on MDSCs

**Figure 3:**
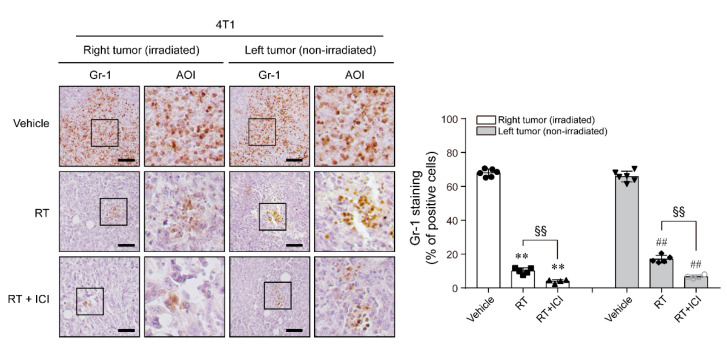
Combined PD-1 blockade therapy and RT regulated the infiltration of MDSCs, showing an abscopal effect. Six-week-old female balb/c nude mice injected with 4T1 cells were sacrificed on day 21 after injection (n = 4–6), and then tumor tissues were obtained and immunohistochemically stained with antibody for GR-1, an MDSC marker. GR-1-positive cells were identified by counting the number of cells stained intensely in fields from tumor tissues of each indicated group. ** *p* < 0.01 compared to the primary (R) tumor of vehicle-treated 4T1-injected group; ^##^ *p* < 0.01 compared to the secondary (L) tumor of vehicle-treated 4T1-injected group; ^§§^ *p* < 0.01; AOI, area of interest; scale bar: 100 μm.

To describe the mechanism underlying the abscopal effect of RT and ICI, we obtained 4T1 tissues and stained GR-1, the marker for MDSCs, after combined RT and ICI treatment (Figure 3). The results showed that the level of GR-1-positive stained cells decreased in both primary and secondary tumors after RT alone or combined RT and ICI treatment. In addition, the number of GR-1-positive stained cells was lower in the combined RT and ICI treatment group than in the RT alone group.

### 2.4. Toxicity

**Figure 4:**
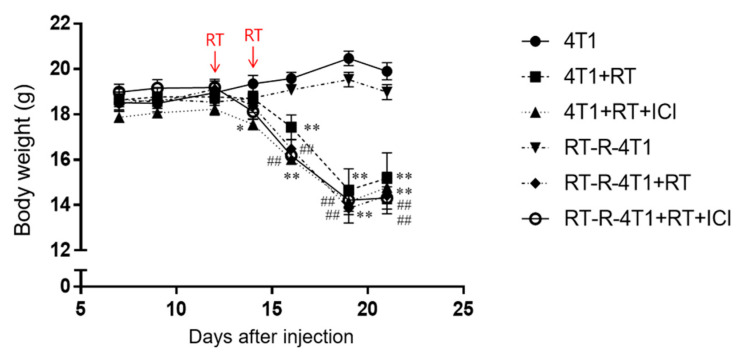
Combined PD-1 blockade therapy and RT showed side effects in the body weights of mice. From day 7 after 4T1 or RT-R-4T1 cancer cell inoculation (n = 4–6), the body weight of mice was measured 3 times a week during tumor development. Arrows indicate the points at which RT was delivered. * *p* < 0.05, ** *p* < 0.01 compared to the 4T1-injected group; ^##^ *p* < 0.01 compared to the RT-R-4T1-injected group.

The body weights of all treated mice decreased immediately after receiving RT and ICI treatment (Figure 4). Notably, in the control group, we did not observe any weight loss, regardless of radiation sensitivity.

## 3. Discussion

In this preclinical study, we investigated the combined effect of local RT and PD-1 blockade therapy for the treatment of metastatic TNBC using a mouse model that closely mimics human TNBC. Our experiments showed that, at least in the relatively radiosensitive 4T1 mammary carcinoma model, the combination of local RT with anti-PD-1 antibody treatment enhanced systemic antitumor effects capable of controlling tumor growth at a remote site consistent with the definition of an abscopal effect. Our data provide the proof of concept that the combination therapy can elicit therapeutically significant antitumor immunity in mice with metastatic 4T1 cancers. 

Although only local tumor control was observed after treatment with RT and ICI in radiation-resistant cell lines, we intend to develop novel treatment strategies for radiation-resistant TNBC patients. 

It is important to note that, although anti-PD-1 antibody administered alone has been shown to induce tumor regression and growth control in other experimental mouse tumors, in our experimental model, the addition of anti-PD-1 antibody to local RT increased the inhibitory effect on the growth of primary or secondary tumors. The abscopal effect was also observed when local RT was delivered alone in radiosensitive tumor cells. This result could be attributed to the radiation dose used in this study. 

Nevertheless, many studies have reported substantial abscopal responses when RT and ICI are combined [22,23,24,25,26]. Combining RT with immunotherapy promotes the cross-priming of tumor-specific CD8^+^ T cells, stimulating the immune effector function of T cells and neutralizing the immunosuppressive effects of the tumor microenvironment [27]. Immunosuppressive cytokines released from tumors or MDSCs, such as transforming growth factor-β (TGF-β) and surface receptors expressed on T cells, such as PD-1 and cytotoxic T-lymphocyte-associated antigen 4 (CTLA-4), can inhibit the function of T cells [28]. Several studies showed that RT inhibits tumor progression by the accumulation of immunosuppressive MDSCs in the tumor microenvironment and exerts its immunosuppressive effect by restraining the activation of T cells [29,30,31]. We observed decreased infiltration of MDSCs in tumors after combining RT and ICI treatment, suggesting that MDSCs play a key role in the abscopal effect.

The expression of PD-L1 is upregulated in tumor cells, and binding of PD-1 with PD-L1 mainly promotes T cell apoptosis and leads to the elimination of activated T cells, thereby protecting tumor cells from T cell recognition and elimination [31]. Although the upregulation of PD-L1 can be observed in experimental mouse tumor models after exposure to hypofractionated RT, the combination of PD-L1 blockade therapy and RT may overcome tumor immunosuppression and improve the systemic effects of RT [20]; as a result, the addition of ICI before resistance to RT in TNBC patients could enhance treatment effectiveness.

We used a radiation dose of 20 Gy in two fractions, and the treatment interval between the irradiations was 2 days. After administration of RT, the mice showed significant and progressive weight loss and slowed movement, indicating that the RT dose used in our study might be too strong to adequately balance the positive effects of irradiation. We did not observe any weight loss in the control group, regardless of radiation sensitivity, suggesting that the dose and interval of RT used in our experiments were toxic to the animal models. Therefore, we need to establish a tolerable and effective treatment plan to establish the appropriate dose of RT, treatment interval of irradiation, and duration of RT and ICI treatment. 

This study has several limitations. We could not estimate the survival of the experimental mice because the radiation dose we used was toxic to them Attempts to adjust the appropriate timing, dosage, location of cancer cell inoculation, and treatment combination were not made; these may be essential for the successful combination RT and ICI treatment. 

## 4. Materials and Methods

### 4.1. Mice

Six-week-old female BALB/c mice were obtained from Orient Bio (Gyeonggi-do, Korea). All experiments were approved by the Institutional Animal Care and Use Committee of Gyeongsang National University (approval number: GNU-200603-M0030; approval date: 3 June 2020).

### 4.2. Cell Line and Reagents

4T1 is a Balb/c-mouse-derived mammary carcinoma cell line. 4T1 cells were grown in RPMI-1640 supplemented with 10% fetal bovine serum and 1% penicillin/streptomycin (all from HyClone; GE Healthcare Life Sciences, Logan, UT, USA). In addition, we repeatedly irradiated 4T1 cells with 2 Gy of radiation 25 times to establish a radiation-resistant cell line, as previously described [32,33]. 

### 4.3. Tumor Challenge and Treatment

**Figure 5:**
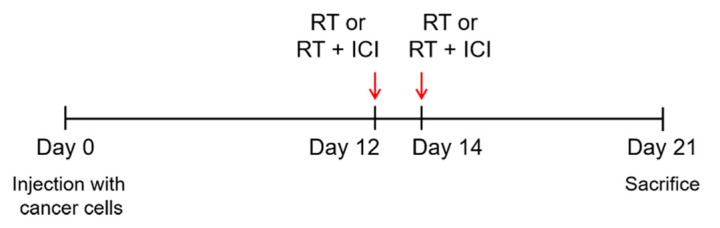
Experimental Schema. Mice were injected s.c. in both flanks with 5 × 10^4^ 4T1 cells on day 0. Treatment was initiated on day 12 from the day of cancer cell inoculation. RT with two fractions of 10 Gy given at a 48 h interval was delivered to a field including the tumor with 5 mm margins. Mice assigned to the ICI group were intraperitoneally administered a fixed dose of 200 µg anti-PD-1 antibody twice at a 48 h interval. All mice were sacrificed on day 21 post-tumor inoculation.

Mice were subcutaneously injected in both flanks with 5 × 10^4^ 4T1 cells in 0.1 mL of RPIM-1640 without additives (n = 4–6). Tumor development was analyzed daily, and the tumor sizes were measured 3 times a week. Tumor volumes were measured using the following formula: tumor volume (mm3) = 4/3 × π × width/2 × depth/2 × height/2. To examine whether anti-PD-1 antibody (pembrolizumab, A2005, Selleckchem, Houston, TX, USA) may influence the RT-induced abscopal effect, 4T1 mammary cancer cells were injected into both the right and left flanks. The right injected tumor cells were irradiated, and the primary tumor cell control was evaluated from these cells; the left-side tumor cells were completely blocked during irradiation, and the secondary tumor regression effect, referred to as the abscopal effect, was investigated from these cells. Twelve days after tumor cell injection, we categorized mice into three groups: (1) control (no treatment with RT or immune checkpoint inhibitor (ICI)), (2) RT alone, and (3) RT+ICI. In RT alone and RT+ICI groups, a single dose of 10 Gy was delivered only to the right-side tumor cells, and then 2 days later, the same dose was re-irradiated to the same site. Pembrolizumab, an ICI, was intraperitoneally injected at 200 µg fixed-dose in mice assigned to the ICI groups before RT administration on the same day. The mice assigned to irradiation were positioned on a flat table with their tumor facing upward under anesthesia by intramuscular injection of zoletil (60 mg/kg). The radiation field was set to include the right-side tumor burden with a 5-mm margin (Figure 5). The rest of the body, other than the irradiated site, was shielded by a metal structure made of ~7-cm-thick tungsten.

Tumor growth was evaluated every 2 or 3 days until the mice died or were sacrificed. All mice were sacrificed on day 21 post-tumor inoculation, and the incidence of lung metastasis was examined by counting the number of metastatic foci on the lung surface after sacrifice.

### 4.4. Immunohistochemical Staining for Infiltrated Myeloid-derived Suppressor Cells (MDSC) Cells in Tumor Tissues

The tumor tissues from mice were fixed in 10% formalin, followed by paraffin infiltration and embedding. Five-micrometer-thick sections were mounted onto MAS-GP type A Coated Slides (Matsunami, Osaka, Japan), and immunohistochemical analysis was performed using anti-GR-1 primary antibodies (RB6-8C5, 1:50, BioLegend, San Diego, CA, USA). Although GR-1+ cells could not define functional specific MDSCs sufficiently, GR-1+ cells are considered myeloid cell markers representing total MDSCs in cancer cells [34]. Horseradish peroxidase-conjugated secondary antibodies were used, and then immunohistochemical staining was performed using an ABC HRP kit (Vector Labs, Burlingame, CA, USA) and diaminobenzidine (DAB) according to the manufacturer’s instructions. Following DAB staining, the sections were counterstained with Mayer’s hematoxylin solution (Cancer Diagnostics, Durham, NC, USA) and observed under a light microscope.

### 4.5. Statistical Analyses

All statistical analyses were performed using the GraphPad Prism software (version 5.0; GraphPad Software Inc., San Diego, CA, USA). One-way ANOVA followed by Tukey’s post hoc test was carried out to compare different groups. The data are presented as the mean ± standard deviation (SD). Statistical significance was set at *p* < 0.05.

## 5. Conclusions

In conclusion, our results revealed that the combination of anti-PD-1 blockade therapy and local RT could lead to the systemic control of tumors. The combination of RT with immunotherapy could promote cancer treatment by harnessing the potential of the immune system in a synergistic manner. Therefore, this study could provide insight into the rational design of combination therapies involving anti-PD-1 and RT to improve responses in patients with primary and metastatic TNBC.

## Data Availability

The data presented in this study are available upon request.

## References

[1] Brenton J., Carey L.A., Ahmed A.A., Caldas C. (2005). Molecular Classification and Molecular Forecasting of Breast Cancer: Ready for Clinical Application?. J. Clin. Oncol..

[2] Banerjee S., Reis-Filho J.S., Ashley S., Steele D., Ashworth A., Lakhani S., Smith I.E. (2006). Basal-like breast carcinomas: Clinical outcome and response to chemotherapy. J. Clin. Pathol..

[3] Bauer K.R., Brown M., Cress R.D., Parise C.A., Caggiano V. (2007). Descriptive analysis of estrogen receptor (ER)-negative, progesterone receptor (PR)-negative, and HER2-negative invasive breast cancer, the so-called triple-negative phenotype: A population-based study from the California cancer Registry. Cancer.

[4] Dent R., Trudeau M., Pritchard K.I., Hanna W.M., Kahn H.K., Sawka C.A., Lickley L.A., Rawlinson E., Sun P., Narod S.A. (2007). Triple-Negative Breast Cancer: Clinical Features and Patterns of Recurrence. Clin. Cancer Res..

[5] Rakha E.A., El-Sayed M.E., Green A., Lee A.H.S., Robertson J.F., Ellis I.O. (2006). Prognostic markers in triple-negative breast cancer. Cancer.

[6] Papadimitriou M., Mountzios G., Papadimitriou C.A. (2018). The role of PARP inhibition in triple-negative breast cancer: Unraveling the wide spectrum of synthetic lethality. Cancer Treat. Rev..

[7] Banerji S., Cibulskis K., Rangel-Escareno C., Brown K.K., Carter S.L., Frederick A.M., Lawrence M.S., Sivachenko A.Y., Sougnez C., Zou L. (2012). Sequence analysis of mutations and translocations across breast cancer subtypes. Nat. Cell Biol..

[8] Mittendorf E.A., Philips A.V., Meric-Bernstam F., Qiao N., Wu Y., Harrington S., Su X., Wang Y., Gonzalez-Angulo A.M., Akcakanat A. (2014). PD-L1 Expression in Triple-Negative Breast Cancer. Cancer Immunol. Res..

[9] Schmid P., Adams S., Rugo H.S., Schneeweiss A., Barrios C.H., Iwata H., Diéras V., Hegg R., Im S.-A., Wright G.S. (2018). Atezolizumab and Nab-Paclitaxel in Advanced Triple-Negative Breast Cancer. N. Engl. J. Med..

[10] Cortes J., Cescon D.W., Rugo H.S., Nowecki Z., Im S.-A., Yusof M.M., Gallardo C., Lipatov O., Barrios C.H., Holgado E. (2020). Pembrolizumab plus chemotherapy versus placebo plus chemotherapy for previously untreated locally recurrent inoperable or metastatic triple-negative breast cancer (KEYNOTE-355): A randomised, placebo-controlled, double-blind, phase 3 clinical trial. Lancet.

[11] Adams S., Schmid P., Rugo H.S., Winer E.P., Loirat D., Awada A., Cescon D.W., Iwata H., Campone M., Nanda R. (2019). Pembrolizumab monotherapy for previously treated metastatic triple-negative breast cancer: Cohort A of the phase II KEYNOTE-086 study. Ann. Oncol..

[12] Hanna G.G., Coyle V.M., Prise K. (2015). Immune modulation in advanced radiotherapies: Targeting out-of-field effects. Cancer Lett..

[13] Postow M.A., Callahan M.K., Barker C.A., Yamada Y., Yuan J., Kitano S., Mu Z., Rasalan T., Adamow M., Ritter E. (2012). Immunologic Correlates of the Abscopal Effect in a Patient with Melanoma. N. Engl. J. Med..

[14] Dagoglu N., Karaman S., Caglar H.B., Oral E.N. (2019). Abscopal Effect of Radiotherapy in the Immunotherapy Era: Systematic Review of Reported Cases. Cureus.

[15] Van Limbergen E.J., De Ruysscher D.K., Olivo Pimentel V., Marcus D., Berbee M., Hoeben A., Rekers N., Theys J., Yaromina A., Dubois L.J. (2017). Combining radiotherapy with immunotherapy: The past, the present and the future. Br. J. Radiol..

[16] Demaria S., Coleman C.N., Formenti S.C. (2016). Radiotherapy: Changing the Game in Immunotherapy. Trends Cancer.

[17] Demaria S., Golden E.B., Formenti S.C. (2015). Role of Local Radiation Therapy in Cancer Immunotherapy. JAMA Oncol..

[18] De Ruysscher D., Niedermann G., Burnet N.G., Siva S., Lee A.W., Hegi-Johnson F. (2019). Radiotherapy toxicity. Nat. Rev. Dis. Primers.

[19] Grass G.D., Krishna N., Kim S. (2016). The immune mechanisms of abscopal effect in radiation therapy. Curr. Probl. Cancer.

[20] Deng L., Liang H., Burnette B., Beckett M., Darga T., Weichselbaum R.R., Fu Y.-X. (2014). Irradiation and anti–PD-L1 treatment synergistically promote antitumor immunity in mice. J. Clin. Investig..

[21] Le H.K., Graham L., Cha E., Morales J.K., Manjili M.H., Bear H.D. (2009). Gemcitabine directly inhibits myeloid derived suppressor cells in BALB/c mice bearing 4T1 mammary carcinoma and augments expansion of T cells from tumor-bearing mice. Int. Immunopharmacol..

[22] Ko Y., Jin H., Lee J., Park S., Chang K., Kang K., Jeong B., Kim H. (2018). Radioresistant breast cancer cells exhibit increased resistance to chemotherapy and enhanced invasive properties due to cancer stem cells. Oncol. Rep..

[23] Jin H., Rugira T., Ko Y.S., Park S.W., Yun S.P., Kim H.J. (2020). ESM-1 Overexpression is Involved in Increased Tumorigenesis of Radiotherapy-Resistant Breast Cancer Cells. Cancers.

[24] Bronte V., Brandau S., Chen S.-H., Colombo M.P., Frey A.B., Greten T.F., Mandruzzato S., Murray P.J., Ochoa A., Ostrand-Rosenberg S. (2016). Recommendations for myeloid-derived suppressor cell nomenclature and characterization standards. Nat. Commun..

[25] Ngwa W., Irabor O.C., Schoenfeld J.D., Hesser J., Demaria S., Formenti S.C. (2018). Using immunotherapy to boost the abscopal effect. Nat. Rev. Cancer.

[26] Liu Y., Dong Y., Kong L., Shi F., Zhu H., Yu J. (2018). Abscopal effect of radiotherapy combined with immune checkpoint inhibitors. J. Hematol. Oncol..

[27] Zhao X., Shao C. (2020). Radiotherapy-Mediated Immunomodulation and Anti-Tumor Abscopal Effect Combining Immune Checkpoint Blockade. Cancers.

[28] DeMaria S., Kawashima N., Yang A.M., Devitt M.L., Babb J., Allison J., Formenti S.C. (2005). Immune-mediated inhibition of metastases after treatment with local radiation and CTLA-4 blockade in a mouse model of breast cancer. Clin. Cancer Res..

[29] Jutzy J.M.S., Lemons J.M., Luke J.J., Chmura S.J. (2018). The Evolution of Radiation Therapy in Metastatic Breast Cancer: From Local Therapy to Systemic Agent. Int. J. Breast Cancer.

[30] Barker H.E., Paget J.T.E., Khan A., Harrington K. (2015). The tumour microenvironment after radiotherapy: Mechanisms of resistance and recurrence. Nat. Rev. Cancer.

[31] Vatner R.E., Cooper B.T., Vanpouille-Box C., DeMaria S., Formenti S.C. (2014). Combinations of Immunotherapy and Radiation in Cancer Therapy. Front. Oncol..

[32] Yin Z., Li C., Wang J., Xue L. (2019). Myeloid-derived suppressor cells: Roles in the tumor microenvironment and tumor radiotherapy. Int. J. Cancer.

[33] Vatner R.E., Formenti S.C. (2015). Myeloid-Derived Cells in Tumors: Effects of Radiation. Semin. Radiat. Oncol..

[34] Chen H.M., Ma G., Gildener-Leapman N., Eisenstein S., Coakley B.A., Ozao J., Mandeli J., Divino C., Schwartz M., Sung M. (2015). Myeloid-Derived Suppressor Cells as an Immune Parameter in Patients with Concurrent Sunitinib and Stereotactic Body Radiotherapy. Clin. Cancer Res..

